# Arrhythmic Risk in Elderly Patients Candidates to Transcatheter Aortic Valve Replacement: Predictive Role of Repolarization Temporal Dispersion

**DOI:** 10.3389/fphys.2019.00991

**Published:** 2019-08-06

**Authors:** Gianfranco Piccirillo, Federica Moscucci, Marcella Fabietti, Ilaria Parrotta, Fabiola Mastropietri, Claudia Di Iorio, Teresa Sabatino, Davide Crapanzano, Giulia Vespignani, Marco Valerio Mariani, Nicolò Salvi, Damiano Magrì

**Affiliations:** ^1^Dipartimento di Scienze Cardiovascolari, Respiratorie, Geriatriche, Anestesiologiche e Nefrologiche, Policlinico Umberto I, “La Sapienza” University of Rome, Rome, Italy; ^2^Dipartimento di Medicina Clinica e Molecolare, S. Andrea Hospital, “Sapienza” University of Rome, Rome, Italy

**Keywords:** aortic stenosis, TAVR, QT, QT standard deviation, T peak-T end, QTc, QT variability

## Abstract

**Background/Aim:**

Degenerative aortic valve stenosis (AS) is associated to ventricular arrhythmias and sudden cardiac death, as well as mental stress in specific patients. In such a context, substrate, autonomic imbalance as well as repolarization dispersion abnormalities play an undoubted role. Aim of the study was to evaluate the increase of premature ventricular contractions (PVC) and complex ventricular arrhythmias during mental stress in elderly patients candidate to the transcatheter aortic valve replacement (TAVR).

**Methods:**

In eighty-one elderly patients with AS we calculated several short-period RR- and QT-derived variables at rest, during controlled breathing and during mild mental stress, the latter being represented by a mini-mental state evaluation (MMSE).

**Results:**

All the myocardial repolarization dispersion markers worsened during mental stress (*p* < 0.05). Furthermore, during MMSE, low frequency component of the RR variability increased significantly both as absolute power (LF_RR_) and normalized units (LF_RRN__U_) (*p* < 0.05) as well as the low-high frequency ratio (LF_RR_/HF_RR_) (*p* < 0.05). Eventually, twenty-four (30%) and twelve (15%) patients increased significantly PVC and, respectively, complex ventricular arrhythmias during the MMSE administration. At multivariate logistic regression analysis, the standard deviation of QTend (QTe_sd_), obtained at rest, was predictive of increased PVC (odd ratio: 1.54, 95% CI 1.14–2.08; *p* = 0.005) and complex ventricular arrhythmias (odd ratio: 2.31, 95% CI 1.40–3.83; *p* = 0.001) during MMSE. The QTe_sd_ showed the widest sensitive-specificity area under the curve for the increase of PVC (AUC: 0.699, 95% CI: 0.576–0.822, *p* < 0.05) and complex ventricular arrhythmias (AUC: 0.801, 95% CI: 0.648–0.954, *p* < 0.05).

**Conclusion:**

In elderly with AS ventricular arrhythmias worsened during a simple cognitive assessment, this events being a possible further burden on the outcome of TAVR. QTe_sd_ might be useful to identify those patients with the highest risk of ventricular arrhythmias. Whether the TAVR could led to a QTe_sd_ reduction and, hence, to a reduction of the arrhythmic burden in this setting of patients is worthy to be investigated.

## Introduction

Senile degenerative aortic valve stenosis (AS) represents the most relevant valvular heart disease both in terms of prevalence and of prognostic implications in Western countries. Indeed, about 3.4% of over 75 years subjects suffers from this valvulopathy (Osnabrugge et al., 2013) and, after the beginning of symptoms, in absence of surgical or transcatheter replacement, the survival is less than half at 2 years (Lindman et al., 2014; Afilalo et al., 2017). Obviously, the poor prognosis in this setting of patients is strongly influenced by a number of possible comorbidities over the AS. However, a mainly neglected factor possibly impacting the AS patients prognosis is represented by their propensity to the malignant arrhythmias. Myocardial hypertrophy, fibers disarray, fibrosis, necrosis and calcification are all features constituting an optimum structural substrate for arrhythmic sudden cardiac death. Furthermore, sympathetic over-activity, typical in chronic heart failure, could also play an important role as malignant ventricular arrhythmias’ trigger. In such a context, there are two previous observations corroborating these claims: it was recently confirmed that the sudden cardiac death during AS remains statistically important (Minamino-Muta et al., 2017) and, some non-invasive electrocardiographic (ECG) markers were found significantly associated to a poor outcome in elderly patients with AS after the transcatheter aortic valve replacement (TAVR) (Piccirillo et al., 2018).

Therefore, the present study evaluated a number of non-invasive markers of myocardial electrical instability in a cohort of elderly patients with AS candidate to the TAVR procedure. Particularly, we analyzed the short period RR- and QT-interval variables (Baumert et al., 2016) at rest, during controlled breathing and during mild mental stress, the latter being represented by a mini-mental state evaluation (MMSE). Thereafter we evaluated a possible MMSE-induced increase in premature ventricular contraction (PVC) or complex ventricular arrhythmias (bigeminy, trigeminy, couplets episodes, R on T phenomena, sustained or non-sustained ventricular tachycardia) (Zanobetti et al., 2017). Eventually, we sought to assess a possible relationship between the abovementioned ECG derived markers obtained during rest and the arrhythmic risk in terms of complex ventricular arrhythmias increase during MMSE.

The major part of these repolarization markers are normalized for RR variability (Baumert Europace 2016; 18, 925–944) (Baumert et al., 2016) and for this reason the patients with frequent premature contractions or atrial fibrillation are frequently excluded from these kind of studies. Notwithstanding, the elderly with AS presented a very high level of supra- or ventricular arrhythmias, consequently we decided to use repolarization indexes only, without RR variability normalization; in this way, we were able to include even patients with atrial fibrillation or with frequent premature atrial or ventricular contractions.

## Materials and Methods

### Participants and Protocol

A total of 92 consecutive symptomatic (NYHA III class) elderly patients who underwent evaluation for TAVR HCM were recruited between September 2017 and July 2018 at Policlinico Umberto I University Hospital in Rome. Patients’ characteristics, preoperative echocardiographic issues, a complete functional assessment and ECG-derived data were recorded at time of enrollment.

The functional assessment included the following: Mini-Mental State Examination (MMSE), Activity of Daily Living (ADL), Instrumental Activities of Daily Living (IADL), and Mini-Nutritional Assessment (MNA). Furthermore the Clinical Frailty Scale (Rockwood et al., 2005) and the Essential Frailty Toolset (Afilalo et al., 2017) have been administered.

The ECG study included, for each patients, three distinct and consecutive sessions with a short-period single lead (II) ECG acquired in supine position: the first session during rest (REST); the second session during controlled breathing (15 breaths per minute) (RESP) and the third one during MMSE (MENTAL STRESS). Both the REST and RESP recordings lasted 5 min while the MENTAL STRESS session lasted averagely 10 min (11.5 ± 3.9 min), being the sum of the three recordings equal to 22.1 ± 3.9 min. Contextually, a non-invasive beat-to-beat blood pressure wave recordings (Finometer MIDI, FMS B.V., Amsterdam, Netherlands) has been recorded.

No patient has been excluded from the ECG analysis, being included also those with atrial and ventricular arrhythmias (premature ventricular or atrial contractions, atrial fibrillation, etc.) or pacemaker. Concerning the latter category, the pacing setting during the study was VDD with lower rate well below the patient’s lowest intrinsic heart rate so that the physiological atrial tracking under study conditions has been preserved. In patients with bundle branch block, J-T interval was considered in place of QT.

The study was approved by the Ethical Committee of Azienda Universitaria Policlinico Umberto I. Each patients signed an appropriate informed consent. Trial was registered on ClinicalTrial.gov database with number NCT03145376.

### Off-Line Data Analysis

To acquire and digitalize the ECG and pressure signals, we used a custom-designed card (National Instruments USB-6008; National Instruments, Austin, TX, United States) with a sampling frequency equal to 500 Hz. The software for data acquisition, storage, and analysis with the LabView program (National Instruments), designed and produced from our research team, follows the technical recommendation of consensus guidance endorsed by European Heart Rhythm Association jointly with the European Society of Cardiology Working Group on cardiac cellular electrophysiology (Baumert et al., 2016). With respect the QT-derived measurements, they were obtained with the template method proposed by Berger et al. (1997).

**FIGURE 1:**
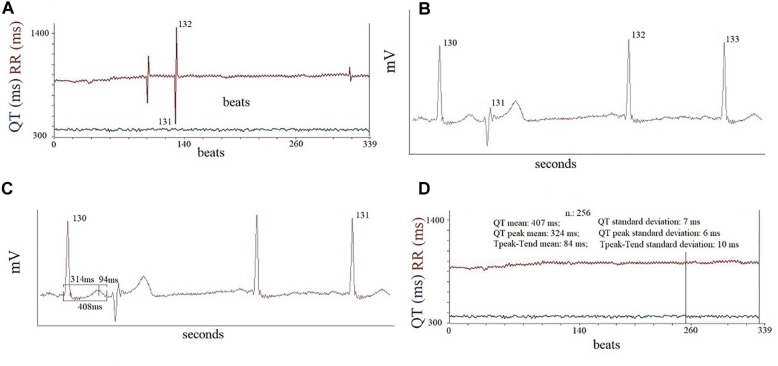
An example of RR and QTe recordings at rest **(A)**, it is possible to observe three PVC in picture, the second PVC is the beat number 131. In the second phase of off-line analysis **(B)**, the computer eliminated the data of PVC (131) and of following beats number 132. Note, the complete flattening of T wave of beat number 132. Therefore, this beats was eliminated from final analysis of data **(C)**. Finally, it was reported the some final analyses in the 256 window data recordings **(D)**.

**FIGURE 2:**
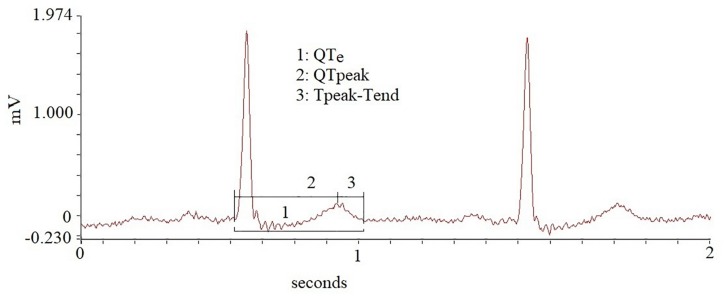
Different repolarization intervals obtained in the study.

Each ECG recording undergoes three consecutive processes: rhythm analysis; elimination of ventricular and atrial premature contraction (PVC ad sPVC) from ECG traces; RR and QT interval analysis. During the rhythm analysis, a quantitative evaluation of PVC has been made by dividing the number of PVC every 3 min of each single examined recording thus disclosing the patients with the increase of PVC per minutes during the MENTAL STRESS session. If during the MENTAL STRESS session only, patients showed bigeminy, trigeminy, couplets episodes, R on T phenomenon, sustained or non-sustained ventricular tachycardia, we considered this fact as an increase of arrhythmias (Zanobetti et al., 2017). Secondly, we identified the PVC and sPVC on the traces and we eliminated manually their QRS-T data and also the corresponding following beat (Figure 1), as recommended in previous consensus guidance (Baumert et al., 2016). After this preliminary phase, we used three time-series of “cleaned” 256 consecutive QRS-T (REST, RESP, and MENTAL STRESS) to study the repolarization variables. With respect the MENTAL STRESS recording, we focused on the period with the higher sympathetic activity (i.e., the QRS-T data series with lower RR cycle length and then higher heart rate). Short-term myocardial temporal repolarization dispersion measurements were obtained on three different intervals: the interval from Q to end of T wave (QTe); the interval between and the Q and the peak of T wave (QTp); the interval between peak and end of T wave (Te) (Figure 2). We then calculated the following QT-derived data: mean and standard deviation of QTe, QTp and Te (QTe_m_, QTe_sd_, QTp_m_, QTp_sd_, Te_m_, and Te_sd_), Te_m_ and QTe_m_ ratio (Te_m_/QTe_m_). We also calculated normalized QTe (QTeVN), QTp (QTpVN) and Te (TeVN) interval variances (Baumert et al., 2016) according to the formulas:

QTeVN=QTe/sd2QeT;m2

QTpVN=QTp/sd2QTp;m2

TeVN=Te/sd2Te.m2

Short term variability of QTe (QTe_STV_), QTp (QTp_STV_) and Te (Te_STV_) (Baumert et al., 2016) was also obtained according to the formulas:

QTe=STVΣ[QT-n+1QT]n(256×√2);

QTp=STVpΣ[QTp-n+1QTp]n(256×√2);

Te=STV[Te-n+1Te]n(256×√2).

Furthermore, we calculated the spectral coherence between the QTp and Te (Piccirillo et al., 2014b) on the 256 beats in the three different study sessions according to the formula (Baumert et al., 2016):

$${\text{Coherence}}_{\left(\text{QTp}-\text{Te}\right)}^{2}\,\left(f\right);$$

$$\frac{\begin{array}{c} \mathrm{Cross}\,\mathrm{spectral}\,\mathrm{power}\,{\mathrm{density}}_{\left(\text{QTp}-\text{Te}\right)}^{2}\,\left(f\right)\\ =|{\text{Coherence}}_{\left(\text{QTp}-\text{Te}\right)}{\left(f\right)}^{2} \end{array}}{\mathrm{Spectral}\,{\mathrm{density}}_{\text{Te}}\,\left(f\right)\,\mathrm{Spectral}\,{\mathrm{density}}_{\text{QTp}}\,\left(f\right)}$$

where *f* was the spectral frequency.

The spectral coherence value ranges between 0 and 1 with the high level of coherence being closer to 1 and it indicates the temporal relation between two signals (QTp and Te).

We also measured manually, by means an electronic caliper and applying the tangent method on three consecutive cycles (II lead), the following intervals: QT (from q to end T wave); QRS (from q to end S wave); JT (from J point to end of T wave); Te (from the peak to the end of T wave) and we corrected them on three preceding RR interval with Bazett method (QT_Bazett_: QT/RR^0.5^; QRS_Bazett_: QRS/RR^0.5^; JT_Bazett_: JT/RR^0.5^; Te_Bazett_/RR^0.5^) (Crow et al., 2003; Rautaharju et al., 2004, 2009).

Eventually, only in those patients with sinus rhythm, excluding those with higher than one PVC per minute, we obtained the spectral and cross-spectral analysis, using the autoregressive method (Task Force of the European Society of Cardiology, and the North American Society of Pacing, and Electrophysiology, 1996; Berger et al., 1997; Baumert et al., 2016) and we reported the following RR and systolic blood pressure (SBP) variables: the total power (TP_RR_, TP_SBP_), resulted from the spectral densities included between the 0 and 0.40 Hz; the high-frequency (HF_RR_, HF_SBP_) component (from 0.15 to 0.40 Hz); the low-frequency (LF_RR_, LF_SBP_) component (from 0.04 to 0.15 Hz Eq); the very-low frequency (VLF_RR_, VLF_SBP_) component (below 0.04 Hz Eq) (Task Force of the European Society of Cardiology, and the North American Society of Pacing, and Electrophysiology, 1996; Piccirillo et al., 2009b, 2016). We also calculated the LF (LF_NU_) and HF (HF_NU_) normalized units according the following formulas:

LF=NULF/RR(TP-RRVLF)RR×100;

HF=NUHF/RR(TP-RRVLF)RR×100.

We also measured LF and HF central frequencies and the α index was calculated according to the formulas: (Robbe et al., 1987; Pagani et al., 1988; Piccirillo et al., 2000a,b, 2004b, 2013, 2016).

αLF=√LF/RR√LF;SBP

αHF=√HF/RR√HF.SBP

Absolute power, LF/HF, α LF and α HF were converted in natural logarithm (ln) (Task Force of the European Society of Cardiology, and the North American Society of Pacing, and Electrophysiology, 1996; Piccirillo et al., 2009b, 2016).

### Statistical Analysis

All data with normal distribution were expressed as means ± standard deviation; non-normally distributed variables were expressed as median and inter-quartile range (iqr); categorical variables are presented as frequencies and percentage (%). In normal distributed data one-way repeated-measures ANOVA test has been used to compare the same variable in the three different study session (REST, RESP, and MENTAL STRESS); the variables with non-normal distribution were compared using Friedman test.

Then, we grouped patients in two categories according to the presence or the absence of complex ventricular arrhythmias recorded during the MENTAL STRESS session. The criteria to include a patient in the complex ventricular arrhythmia group were the following rhythm disturbs during the MENTAL STRESS session: bigeminy, trigeminy or couplet episodes, R on T phenomenon, sustained or non-sustained ventricular tachycardia (Zanobetti et al., 2017). Thereafter we also grouped the patients in two other categories according the presence or the absence of an increase PVC per minute during the MENTAL STRESS session. The values of repolarization obtained during REST of these study groups were compared using Student’s T and Mann-Whitney U tests, respectively for normal and non-normal distribution data.

Uni- and multivariable forward (A. Wald) stepwise logistic regression analysis were used to determine the association between the increase of the number or the complex ventricular arrhythmias during the MENTAL STRESS session and clinical, hemodynamic, repolarization and spectral data during the REST session. Particularly, we considered covariates the following repolarization data: QTe_m_, QTe_sd_, QTp_m_, QTp_sd_, Te_m_, Te_sd_, Te_m_/QTe_m_, QTe_STV_, QTp_STV_, Te_STV_, Coherence_(QTp–Te)_,QRS, QT, JT, Te, QRS_Bazett_, QT_Bazett_, JT_Bazett_, Te_Bazett_, Te/QTe, Te_Bazett_/QTe_Bazett_. QTeVN, QTpVN, TeVN were excluded from the present analysis because of their non-normal distribution. Receiver operating characteristic (ROC) curves were used to determine the sensitivity and specificity of studied parameters predictive of complex ventricular arrhythmias and areas under ROC curves and 95% confidence intervals (CI) were calculated to compare the diagnostic efficiencies. All data were evaluated by use of database SPSS-PC + (SPSS-PC + Inc., Chicago, IL, United States).

## Results

From the initial 92 patients’ study sample, 11 patients were excluded because the ECG traces’ poor quality (No. 4 patients) or because they did not complete the protocols (No. 6 patients). Table 1 summarized clinical, echocardiographic, cognitive, nutritional and functional data for a total of 81 elderly patients effectively enrolled in the present study.

**TABLE 1:** General characteristic of the degenerative aortic valve stenosis.

	**N: 81**
Age, years	81 ± 7
M/F,	36/45
BMI, kg/m^2^	26.7 ± 4.5
Complete right bundle branch block	6(7)
Complete left bundle branch block	10(12)
Aortic peak gradient, mm Hg	73 ± 23
Aortic mean gradient, mm Hg	45 ± 15
Aortic valve area, cm^2^/m^2^	0.46 ± 0.14
Aortic peak velocity, m/s	4.2 ± 0.8
Ejection fraction,%	51 ± 9
Stroke volume index, ml/m^2^	41 ± 17
Left ventricular mass index, g/m^2^	143 ± 39
Mini-mental state evaluation	26.3 ± 3.9
Activity of day living	5 ± 1
Instrumental activities of day living	5 ± 2
Clinical frailty scale	4 ± 1
Essential frailty toolset	2 ± 1
Mini-nutritional assessment	23 ± 4
β-blockers, n (%)	43(53)
Verapamil/Diltiazem, n (%)	4(5)
Amiodarone, n (%)	4(5)
Flecainide, n (%)	2(2)
Propafenone, n (%)	1(1)
Ivabradine, n (%)	2(2)
Digoxin, n (%)	4(5)
ACE/sartan, n (%)	47(58)
Dihydropyridine calcium channel blockers, n (%)	26(32)
Furosemide, n (%)	46(57)
Nitrate, n (%)	7(9)
Ranolazine, n (%)	6(7)
Statine, n (%)	37(46)
Antiplatelet therapy, (%)	39(48)
Oral anticoagulants, (%)	26 (32)
Pacemaker, n (%)	5(6)

*Data are expressed as mean ± SD or number (n) of patients (%).*

The arrhythmic characteristics obtained during the three sessions (REST, RESP, MENTAL STRESS) were reported in the Table 2. During the MENTAL STRESS session, an increase of PVC and of arrhythmic ventricular complexity were found in 24 (from 0.3 [2.1] to 0.8 [3.9], *p* < 0.001) and, respectively, in 12 patients (from 0 [0] to 7 ventricular bigeminy or trigemini – 8 ventricular couplets episodes; 3 non-sustained ventricular tachycardia; 3 R on T phenomenon). Remarkably, two patients with an increased complexity of ventricular arrhythmias during MENTAL STRESS did not report any isolated PVC during REST (both of them showed a ventricular couplet episode and, only one of them, an R on T phenomenon, too). Three subjects showed premature ventricular couplets during the RESP session, these type of arrhythmic episodes interesting a total of 11 patients. No significant difference was found between those patients with increased PVC’s number or complexity of ventricular arrhythmias and all the other AS patients with respect clinical, cognitive, nutritional, functional and echocardiographic data.

**TABLE 2:** Arrhythmic characteristic of study subjects during short term ECG monitoring.

	**N: 81**
Sinus rhythm	59(73)
Permanent atrial fibrillation	22(27)
Premature supraventricular contraction	17(21)
Premature ventricular contraction	50(62)
>1 Premature ventricular contraction/minute	19(23)
<1 Premature ventricular contraction/minute	31(38)
Complex ventricular arrhythmias	15(19)
Ventricular bigeminy or trigeminy	7(9)
Premature ventricular couplets	11(14)
Non-sustained ventricular tachycardia	3(4)
R on T phenomenon	3(4)
Increasing premature ventricular contractions during mental stress	24(30)
Increasing ventricular arrhythmic complexity during mental stress	12(15)

### Hemodynamic and Repolarization Data

During MENTAL STRESS, all patients reported a significant increase of heart rate (*p* < 0.01) and, at the same time, they significantly reduced the non-invasively measured stroke volume (*p* < 0.001) and cardiac output (*p* < 0.05) (Table 3).

**TABLE 3:** Hemodynamic (Fenometer) and short period repolarization variability data obtained on 256 beats in all study subjects.

	**Rest**	**Controlled breathing**	**Mental challenge**	***P*** ***ANOVA***
	**N:81**	**N:81**	**N:81**	
*Variables*				
Heart rate, b/m	69 ± 11^∗∗^	69 ± 11^∗∗^	72 ± 12	<0.001
Systolic blood pressure, mm Hg	119 ± 23	118 ± 24	116 ± 41	Ns
Diastolic blood pressure, mm Hg	62 ± 11	61 ± 12	62 ± 20	Ns
Stroke volume, ml	39 ± 13^∗∗^	39 ± 14^∗∗^	35 ± 18	<0.001
Cardiac output, l/m	2.72 ± 0.94	2.71 ± 0.98^*^	2.48 ± 1.20	0.032
Peripheral resistance, a.u.	3853 ± 2316	3925 ± 2431	4425 ± 3342	Ns
QTe mean, ms	408 ± 53	412 ± 53	407 ± 50	ns
QTe standard deviation, ms	7 ± 2§§^*^	8 ± 2^*^	11 ± 2	<0.001
QTp mean, ms	328 ± 45	326 ± 48	322 ± 45	Ns
QTp standard deviation, ms	7 ± 2^*^	7 ± 2^*^	9 ± 5	0.002
Te mean, ms	80 ± 24§^*^	86 ± 24	85 ± 24	0.026
Te standard deviation, ms	10 ± 2^*^	10 ± 2^*^	13 ± 9	<0.001
Te mean/QTe mean	0.22 ± 0.06§^*^	0.24 ± 0.06	0.24 ± 0.06	0.005
QTeVN	0.28[0.21]§§^∗∗^	0.33[0.33]^∗∗^	0.46[0.29]	<0.001
QTpVN	0.56[0.49]^∗∗^	0.58[0.51]^∗∗^	0.97[2.00]	<0.001
TeVI	14[21.33]^∗∗^	14[15]^*^	21[20]	<0.001
Coherence_(QTp–Te)_^2^	0.600 ± 0.139§^*^	0.555 ± 0.122	0.552 ± 0.115	0.002
QTe_STV_	14 ± 4§§^∗∗^	15 ± 4^*^	19 ± 13	<0.001
QTp_STV_	14 ± 5^*^	15 ± 6^*^	16 ± 6	0.023
Te_STV_	20 ± 6^*^	21 ± 8	25 ± 13	0.010

Values are expressed as mean ± SD or median [interquartile range 75th percentile – 25th percentile]. ^∗∗^*p* < 0.001 REST or RESP vs. MENTAL STRESS; ^*^p < 0.05 REST or RESP vs. MENTAL STRESS; §§*p* < 0.001 REST vs. RESP; §*p* < 0.05 REST vs. RESP.

The QTe mean and QTp_m_ values were steady between REST and MENTAL STRESS session while Te_m_ significantly increased (*p* < 0.05) (Table 3). Moreover the Te_m_ value increased significantly during the RESP session in comparison to the REST one, too. All markers of myocardial ventricular temporal dispersion, excepted the Te_m_/QTe_m_, were significantly higher during the MENTAL STRESS in comparison to the REST (*p* < 0.05) and RESP (*p* < 0.05) (Table 3). Instead, the Coherence_(QTp–Te)__2_ showed a mirrored trend, this variable decreasing during MENTAL STRESS and RESP in comparison to the REST session (*p* < 0.05) (Table 3). Eventually, during the RESP session, in all study patients a significant increase of QTe (*p* < 0.001), Te_m_/QTe_m_ (*p* < 0.05) and QTeVN (*p* < 0.05) in comparison to the REST session have been observed (Table 3).

During MENTAL STRESS, the repolarization data manually obtained were almost steady (Table 4) when corrected for the heart rate (Bazett). Only the Te_Bazett_ value decreased significantly during the MENTAL STRESS with respect the REST and RESP sessions (*p* < 0.001).

**TABLE 4:** Manual repolarization data obtained on 3 QRS-T cycles.

	**Rest**	**Controlled breathing**	**Mental challenge**	***P*** ***ANOVA***
	**N:81**	**N:81**	**N:81**	
*Variables*				
RR, ms	881 ± 150^*^	873 ± 134^*^	853 ± 133	0.017
QT, ms	425 ± 54^*^	424 ± 53^*^	414 ± 49	0.003
QRS, ms	91 ± 23	91 ± 24	93 ± 40	Ns
JT, ms	334 ± 55^*^	333 ± 54^*^	321 ± 64	0.006
Te, ms	92 ± 25^*^	88 ± 20	86 ± 20	0.034
QT_Bazett_, ms	455 ± 48	455 ± 51	450 ± 41	Ns
QRS_Bazett_, ms	98 ± 28	99 ± 29	102 ± 47	Ns
JT_Bazett_, ms	357 ± 49	356 ± 50	348 ± 61	Ns
Te_Bazett_, ms	98 ± 28^∗∗^	95 ± 21^*^	94 ± 23	<0.001
Te/QTe,	0.21 ± 0.5	0.21 ± 0.5	0.21 ± 0.5	Ns
Te_Bazett_/QTe_Bazett_	0.21 ± 0.5	0.21 ± 0.4	0.21 ± 0.4	Ns

Values are expressed as mean ± SD or median [interquartile range 75th percentile – 25th percentile]. ^∗∗^*p* < 0.001 REST or RESP vs. MENTAL STRESS; ^*^p < 0.05 REST or RESP vs. MENTAL STRESS.

### RR Spectra Analysis Data

RR and SBP power and cross spectral analysis were obtained in only 59 patients on sinus rhythm. LF, expressed in absolute and normalized power, and LF/HF were significantly higher during MENTAL STRESS (ln LF_RR_: 4.44 ± 1.35 ms^2^; LF NU: 48 ± 17; ln LF/HF; 0.76 ± 1.13) in comparison to REST (ln LF_RR_: 3.66 ± 1.42, *p* < 0.05; LF_NU_: 39 ± 20, *p* < 0.05; ln LF/HF; 0.22 ± 1.1.52, *p* < 0.05) and RESP (ln LF_RR_: 3.63 ± 1.46, *p* < 0.001; LF_NU_: 34 ± 22, *p* < 0.001; ln LF/HF: -0.14 ± 1.17, *p* < 0.001).

HF_NU_ was significantly lower in REST (HF_NU_: 35 ± 25, *p* < 0.05) and RESP (HF_NU_: 39 ± 22, *p* < 0.001) than during MENTAL STRESS (HF_NU_: 26 ± 18).

Both the α indexes, marker of baroreflex sensitivity, were lower during MENTAL STRESS (α LF: 0.80 ± 0.90; α HF: 0.77 ± 0.91) than REST (α LF: 1.32 ± 0.96, *p* < 0.001; α HF: 1.41 ± 0.96, *p* < 0.001) and RESP (α LF: 1.42 ± 0.79, *p* < 0.001; α HF: 1.44 ± 0.95, *p* < 0.001).

No statistically significant difference have been found between RR variability data obtained at REST and RESP.

### Category With PCVs’ Increase During the MENTAL STRESS Session

**FIGURE 3:**
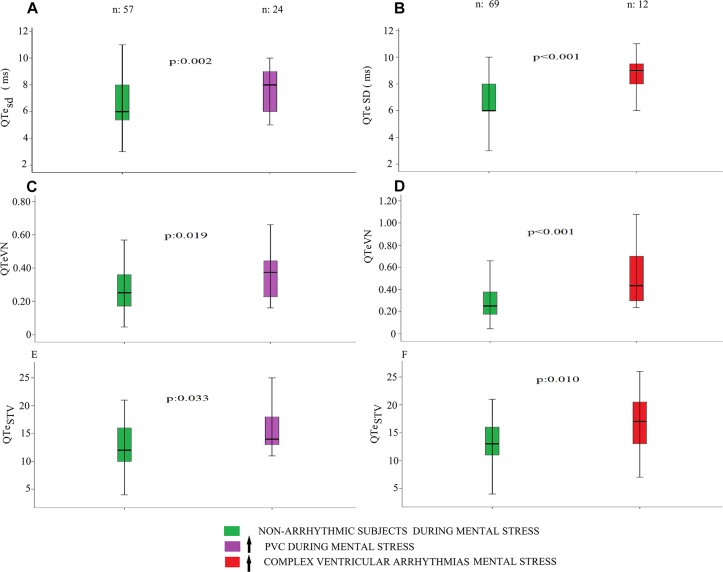
QTe standard deviation QTe_sd_, QTeVN and QTe_STV_, obtained at rest, in subjects with an increase of PVC (purple) and complex ventricular arrhythmias (red) during mental stress. At REST, the 24 patients with an increase of PVC during MENTAL STRESS showed the following repolarization markers significantly higher than other 57 patients: QTe_SD_ (*p* < 0.05), QTeVN (*p* < 0.05), QTe_STV_ (*p* < 0.05) (panel **A**, **C**, and **E**). At REST, the 12 patients with an increased complexity of ventricular arrhythmias during MENTAL STRESS showed the following markers significantly higher than other 69 patients: QTe_sd_ (*p* < 0.001), Te_m_ SD (*p* < 0.05), QTeVN (*p* < 0.001), QTpVN (*p* < 0.05) and QTe_STV_ (*p* < 0.05) (panel **B**, **D**, and **F**).

At REST, the 24 patients with an increase of PVC during MENTAL STRESS showed the following repolarization markers significantly higher than other 57 patients: QTe_SD_ (8 ± 2 vs. 7 ± 2 ms^2^, *p* < 0.05), QTeVN (0.37[0.22] vs. 0.25 [0.20], *p* < 0.05), QTe_STV_ (15 ± 3 vs. 13 ± 5, *p* < 0.05) (Figures 3A,C,E), QRS (102 ± 27 vs. 87 ± 20 ms, *p* < 0.05), QRS_Bazett_ (111 ± 32 vs. 93 ± 25 ms, *p* < 0.05). No other significant differences were observed between these two study groups.

### Category With Complex Ventricular Arrhythmias’ Increase During the MENTAL STRESS Session

At REST, the 12 patients with an increased complexity of ventricular arrhythmias during MENTAL STRESS showed the following markers significantly higher than other 69 patients: QTe_sd_ (9 ± 2 vs. 7 ± 2 ms^2^, *p* < 0.001), Te_m_ SD (12 ± 3 vs. 10 ± 2 ms^2^, *p* < 0.05), QTeVN (0.43[0.49] vs. 0.25 [0.20], *p* < 0.001), QTpVN (0.52[0.56] vs. 0.41 [0.31], *p* < 0.05) and QTe_STV_ (17 ± 5 vs. 13 ± 4, *p* < 0.05) (Figures 3B,D,F). Instead Coherence_(QTp–Te)__2_ was lower in the arrhythmic patients’ group in comparison with the counterpart (0.524 ± 0.119 vs. 0.613, *p* < 0.05). Eventually, excepted the Te/QTe (0.242 ± 0.049 vs. 0.210 ± 0.046 ms, *p* < 0.05) and Te_Bazett_/QTe_Bazett_ (0.241 ± 0.051 vs. 0.210 ± 0.046, *p* < 0.05), most of the manual repolarization indexes were not significantly different between the study groups.

### Relationship Between Ventricular Arrhythmic Risk and Clinical, Hemodynamic and Repolarization Data

Uni- and multivariable logistic regression analysis reported only statistically significant associations between increase of PVC or complex ventricular arrhythmias during MENTAL STRESS and repolarization data at REST (Table 5). None of clinical, echocardiographic, non-invasive hemodynamic spectral data showed a significant relationship with the ventricular arrhythmic risk during the MENTAL STRESS session.

**TABLE 5:** Univariable logistic regression analysis data.

	**↑ PVC during mental stress**	**↑ Complex ventricular arrhythmias during mental stress**
	**Odds ratio (95% CI) *P*-value**	**Odds ratio (95% CI) *P*-value**
QTe standard deviation, ms	1.540(1.114–2.080) *p* = 0.005	2.153(1.338–3.465) *p* = 0.002
Te standard deviation, ms	*p* = ns	1.353(1.061–1.726) *p* = 0.015
Coherence_(QTp–Te)_^2^	*p* = ns	0.009(0–0.930) *p* = 0.047
QTe_STV_	1.131(1.007–1.270) *p* = 0.038	1.207(1.036–1.405) *p* = 0.016
QRS	1.030(1.007–1.053) *p* = 0.010	*p* = ns
QRS_Bazett_	1.022(1.004–1.041) *p* = 0.016	*p* = ns
Te/QTe	*p* = ns	1.143(1.007–1.297) *p* = 0.039
Te_Bazett_/QTe_Bazett_	*p* = ns	1.136(1.002–1.289) *p* = 0.047

The univariable logistic analysis identified the following repolarization variables obtained at REST and the risk factors of PVC increase: QTe_sd_ (*p* < 0.05), QTe_STV_ (*p* < 0.05), QRS (*p* < 0.05), QRS_Bazett_ (*p* < 0.05) (Table 5). On the contrary, the same statistical approach detected the following repolarization variables obtained at REST as predictors of complex ventricular arrhythmias during MENTAL STRESS: QTe_sd_ (*p* < 0.05); Te SD (*p* < 0.05); Coherence_(QTp–Te)__2_ (*p* < 0.05); QTe_STV_ (*p* < 0.05); Te/QTe; Te_Bazett_/QTe_Bazett_ (Table 5).

Multivariable logistic analysis identified only the QTe_SD_ as risk factor of the increase of PVC (odd ratio: 1.54, 95% CI 1.14–2.08; *p* = 0.005) and complex ventricular arrhythmias (odd ratio: 2.31, 95% CI 1.40–3.83; *p* = 0.001).

### Short Period Analysis Versus Manual Measurements: Comparative Study in the Ventricular Arrhythmic Risk Prediction

**FIGURE 4:**
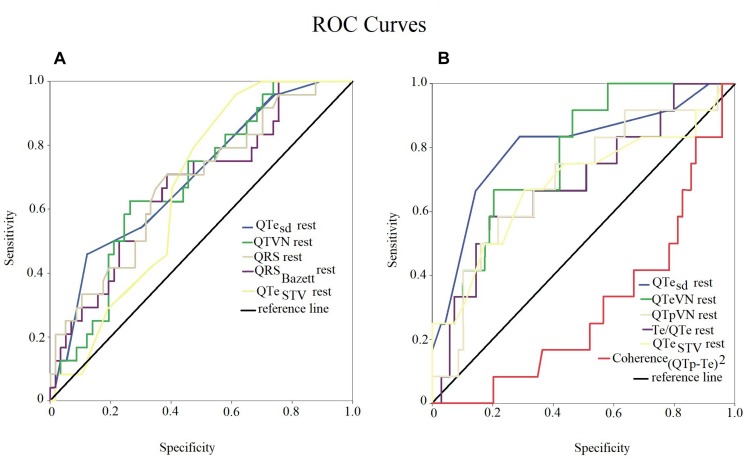
ROC curve of statistical significant examined variables. Sensitivity-specificity of different variables, obtained at rest, to individuate patients with increase of PVC **(A)** and complex ventricular arrhythmias **(B)** during mental stress. QTe standard deviation (QTe_sd_) had the widest area under the curve in both the diagrams (blue lines).

Although several short period and manual repolarization markers reached a sufficient statistical significance only QTe_sd_ showed the widest sensitivity-specificity area under curve (AUC) for predicting both the increase of PVC (AUC: 0.699, 95% CI: 0.576–0.822, *p* < 0.05) and complex ventricular arrhythmias (AUC: 0.801, 95% CI: 0.648–0.954, *p* < 0.05) during the MENTAL STRESS session (Figure 4). Particularly, the other markers with significant area under the curve were: QTeVN (AUC: 0.685, 95% CI 0.565–0.805, *p* < 0.05); QRS (AUC: 0.682, 95% CI 0.556–0.809, *p* < 0.05); QRS_Bazett_ (AUC: 0.673, 95% CI 0.545–0.800, *p* < 0.05); and QTe_STV_ (AUC: 0.664, 95% CI 0.545–0.780, *p* < 0.05) for an increase of PVC during the MENTAL STRESS session (Figure 4A). On the contrary, the other variable with statistically significant area under the curve were: QTeVN (AUC: 0.781, 95% CI 0.655–0.908, *p* < 0.05); QTpVN (AUC: 0.694, 95% CI 0.530–0.859, *p* < 0.05),; Te/QTe (AUC: 0.692, 95% CI 0.525–0.859, *p* < 0.05), QTe_STV_ (AUC: 0.688, 95% CI 0.501–0.875, *p* < 0.05), and Coherence_(QTp–Te)__2_ (AUC: 0.303, 95% CI 0.154–0.451, *p* < 0.05) (Figure 4B).

### Effects of Possible Confounders (β-Blocker Therapy, Atrial Fibrillation)

A concomitant therapy with β-blocker was present in 43 patients (53%) but this group did not any differences with respect the increase PVC or complex ventricular arrhythmias. The others repolarization markers, as well as clinical and hemodynamic data, did not even change in relation to the β-blocker therapy.

Multivariable logistic analysis confirmed QTe_sd_ as predictive of the complex ventricular arrhythmias’ increase also considering sinus rhythm patients alone (odd ratio: 3.17, 95% CI 1.37–7.35; *p* = 0.007), thus excluding from those with atrial fibrillation (odd ratio: 2.92, 95% CI 1.23–6.93; *p* = 0.015). On the contrary, excluding the patients on atrial fibrillation, the same statistical analysis confirmed QTe_sd_ (odd ratio: 1.84, 95% CI 1.59–2.92; *p* = 0.01) predicative only for an increase of PVC during the MENTAL STRESS session.

## Discussion

The main finding of the present study was that a non-negligible percentage of elderly patients with degenerative AS group increased the PVC and complex ventricular arrhythmias during a mild mental stress, such as the one represented by a simple MMSE and, notably, it happens regardless a concomitant beta-blockers therapy. The MMSE is usually needed to assess possible cognitive impairment in elderly candidates to a TAVR procedure (Lindman et al., 2014; Otto et al., 2017). It is highly conceivable that this simple standard test might lead, through a mental arithmetical exercise and several other cognitive tests (orientation, registration recall, language, repetition and complex tasks) (Folstein et al., 1975), to an increase of ventricular arrhythmias due to an increase of sympathetic activity and a reduced vagal tone. Supporting the abovementioned hypothesis, we found a significant increase in the explored sympathetic markers at RR power spectral analysis (ln LF_RR_; LF NU; ln LF/HF) (Task Force of the European Society of Cardiology, and the North American Society of Pacing, and Electrophysiology, 1996; Piccirillo et al., 2009b, 2016) as well a significant reduction in two well-known vagal markers (ln HF_RR_ and ln α HF) (Robbe et al., 1987; Pagani et al., 1988; Piccirillo et al., 2000a,b, 2004b, 2013, 2016).

Another, possibly clinical relevant, finding of the present study was that a simple non-invasive short period myocardial repolarization index, such as the QTe_sd_ obtained at rest, seems to be able to identify those patients with the highest probability to increase ventricular arrhythmias (PVC or complex ventricular arrhythmias) during the MMSE administration. Thus, albeit highly speculative, the QTe_sd_ obtained at rest could be potentially useful in disclosing a general arrhythmias propensity and, accordingly, an increased risk of sudden cardiac death in elderly patients with AS candidate to a TAVR procedure (Massing et al., 2006; Cheriyath et al., 2011; Ataklte et al., 2013; Agarwal et al., 2015; Zanobetti et al., 2017). This clinical feature could frustrate the TAVR’s outcomes and, accordingly, should be worthy to be weighted during the screening procedures. Indeed, although the TAVR improves undoubtedly the hemodynamic patient’s conditions, the myocardial arrhythmic substrate of AS (hypertrophy, disarray, calcifications, ischemia, fibrosis, necrosis, etc.) remains theoretically and practically still able to induce malignant reentrant ventricular arrhythmias also after the AS resolution. In such a context, we also compared the predicative power of conventional QTe and Te measurements with novel short period repolarization variability markers and we found that the QTe_sd_ demonstrated the best accuracy in disclosing those patients more susceptible to increase ventricular arrhythmias during MMSE. Thus, an easy-to-obtain surface ECG-derived parameter, that is the QTe_sd_ obtained at rest, might be considered in guiding at least a more aggressive treatment in these specific category (i.e., high dosage of β-blockers or amiodarone therapy).

### Mental Stress and Sudden Cardiac Death

Emotions are able to trigger malignant ventricular arrhythmias and sudden cardiac death in subjects with known or unknown heart disease and this aspect is particularly relevant in elderly patients. In such a context, retrospective studies highlighted an increase of sudden cardiac death during natural or unnatural thrilling events such as earthquakes (Trichopoulos et al., 1981; Leor et al., 1996; Kario et al., 1997; Kitamura et al., 2013; Kiyohara et al., 2015) bombings (Meisel et al., 1991), terrorist attack (Steinberg et al., 2004) and also football matches (Wilbert-Lampen et al., 2008) or other positive (Phillips et al., 2004) or negative emotional events (Cannon, 2002; Lampert et al., 2002; Williams et al., 2011).

Although no specific data on elderly patients with AS are present in literature, it is easily supposable from a pathophysiologic viewpoint that the simultaneous joint of degenerative valve disease with chronic heart failure and mental stress can exacerbated a tendency for life-threatening arrhythmias. The myocardial hypertrophy and reentrant circuits provide the substratum and electrophysiologic mechanism; the resulting simpato-vagal imbalance, induced by the chronic heart failure, constitutes the “milieu ideal”; and, finally, the sudden sympathetic stimulus, emotion mediated, can easily trigger a fatal arrhythmias. Noteworthy two patients of our study reported ventricular couplets during mental stress without preexistent PVC at rest and one of them reported a R on T phenomenon. Thus it might be hypothesized that the MMSE alone was able to induce a sympathetic overstimulation leading a complex ventricular arrhythmias. Therefore, in these two elderly patients an episode of malignant ventricular arrhythmias could be triggered “like a bolt from the blue” during a high emotional level event. This clinical feature might be clinically relevant in defining the therapeutic strategy: i.e., the elderly patient with AS candidate to a TAVR procedure who shows ventricular complex arrhythmias just during mental stress without any PVC at rest should be aggressively beta-blocked or should receive amiodarone. Clearly, in light of our present data, we strongly recommend the ECG monitoring during MMSE in such patient’s category.

### Sympatho-Vagal Imbalance, Abnormal Repolarization and Ventricular Malignant Arrhythmias

Myocardial repolarization phase is abnormal in patients with myocardial hypertrophy and it might be non-invasively evaluated by analyzing the QT interval prolongation and its dispersion. The molecular basis of these ECG features are complex (Abriel et al., 2015; Rahm et al., 2018). Briefly, in chronic heart failure the potassium channels (I_to_, I_Ks_, I_Kr_ and I_K__1_) are downregulated, sodium channel (I_Na_) shows a delayed inactivation, finally, calcium handling is deeply altered. Then, chronic heart failure is able to induce a prolonged and inhomogeneous action potential duration both in the time and spatial domain, detectable on ECG as a prolonged and temporal dispersed QT interval. This condition constitutes an optimum “pabulum” for reentry arrhythmias. Several experimental and clinical studies, mostly by our research group reported that the sympathetic stimulation was able to exacerbate the QT temporal dispersion in different clinical setting all characterized by myocardial structural abnormalities (Piccirillo et al., 2009a, 2012, 2013, 2014a; Baumert et al., 2016). However, up to now, specific data in elderly patients with AS were not present. Originally we now supplied data with respect a worsening of all markers of myocardial temporal dispersion of repolarization phases during a mild mental stress. In sush a context, between several conventional manually measurements of myocardial repolarization, only Te and Te_Bazett_ were increased during mental stress. Obviously, the temporal dispersion markers were more sensitive to detect the sympathetic-dependent changes, probably because they were obtained on a longer period (256 cycles) in comparison with the manual measurement (3 cycles). These two ECG parameters followed the trend of all short period markers of QT most likely because the Te interval represents the QT interval subsegment more susceptible to the sympathetic variations (Shimizu and Antzelevitch, 2000; Shimizu et al., 2003; Piccirillo et al., 2012, 2013, 2014a). Indeed, in this last part of repolarization phase, I_Ks_ is capable to modulate the QT duration to RR cycle length and, in chronic heart failure, these channels are downregulated. Thus, a mental stress could be sufficient to trigger this alteration also with conventional QT measurement (Aro et al., 2017; Tse et al., 2017; Piccirillo et al., 2018; Yu et al., 2018).

Autonomic cardiovascular regulation is deeply involved in the pathophysiology of the AS, too. The sympathetic drive’s increase and the vagal control alterations are typical of all different stages of this syndrome, together with the baroreflex sensitivity depression. Therefore, RR power spectral analysis shows different spectral pattern according the class impairment and the related therapy. In the first two NYHA classes, the LF spectral component tend to increase (Guzzetti et al., 1995; Yaniv et al., 2014) whereas the most advanced stages are usually associated to a reduction of LF spectral power (Mortara et al., 1994; Guzzetti et al., 1995; Piccirillo et al., 2006, 2009b; Yaniv et al., 2014). The latter changes are also usually observed as quite physiological aging-related changes (Piccirillo et al., 1995, 1998) our sample with symptomatic AS showed a low short period heart rate variability and, consequently LF, in normalize and absolute power, but the patients were still able to increase LF during mental stress; probably this ability could be impaired in comparison with normal age-matched subjects (Piccirillo et al., 1995, 1998). Therefore, the β-blocker treatment can modify all spectral components and LF in particular (Piccirillo et al., 2000a). Nevertheless, chronic heart failure and aging are capable to reduce contextually the HF spectral component (Pagani et al., 1986; Piccirillo et al., 2004a). Eventually, during mental stress, our patients showed a decrease of baroreflex sensitivity indexes (α-index), this behavior mirroring a sympathetic activation and parasympathetic deactivation (Piccirillo et al., 2001a,b; Pinna et al., 2015).

### Temporal Repolarization Variability as Markers of Sudden Cardiac Death

Probably the most dreadful AS complication is sudden cardiac death induced by reentrant ventricular arrhythmias.

## Conclusion

In elderly with AS, ventricular arrhythmias worsened during a simple cognitive assessment, this events could be a further burden on the outcome of TAVR. Although, the TAVR reduces the morbidity and mortality, in some subjects sudden death’s risk remains high. Therefore, it could come in handy to stratify the ventricular malignant arrhythmias risk using a non-invasive, not expensive, repeatable and simple test. In such a context, our data enlightened that QTe_sd_, obtained at rest on 256 consecutive cycles, shows the best accuracy in identifying those patients with AS more prone to develop ventricular arrhythmias. Obviously, in the present study we evaluated the predictive repolarization markers in stratifying the increase of number and complexity of ventricular arrhythmias as a surrogate of major arrhythmic risk (i.e., sudden cardiac death). Then we could reasonably hypothesize that these two markers of electrical ventricular instability could represent the first point of reference waiting more specific data. However, it is reasonable to suggest a more aggressive antiarrhythmic therapy in those patients with AS candidates to TAVR procedures and high QTe_sd_ value at rest.

## Data Availability

The datasets analyzed in this manuscript are not publicly available. Requests to access the datasets should be directed to gianfranco.piccirillo@uniroma1.it.

## Ethics Statement

This study was approved by the Ethical Committee of Azienda Universitaria Policlinico Umberto I. Each patient signed an appropriate informed consent. Trial was registered on ClinicalTrial.gov database with number NCT03145376.

## Author Contributions

GP: conceptualization, data curation, formal analysis, and writing. FeM: writing – review and editing. MF, CDI, FaM, TS, DC, MM, NS, and GV: investigation, methodology, and data curation. IP: data curation. DM: supervision, validation, visualization, review, and editing.

## Conflict of Interest Statement

The authors declare that the research was conducted in the absence of any commercial or financial relationships that could be construed as a potential conflict of interest.
